# QTL analysis of divergent floral morphology traits between *Gilia yorkii* and *G. capitata*

**DOI:** 10.1093/g3journal/jkae106

**Published:** 2024-05-21

**Authors:** Joseph M DeTemple, Daniel H Chitwood, Veronica Mosquera, Clinton J Whipple

**Affiliations:** Department of Biology, Brigham Young University, Provo, UT 84602, USA; Department of Horticulture, Michigan State University, East Lansing, MI 48824, USA; Department of Computational Mathematics, Science & Engineering, Michigan State University, East Lansing, MI 48824, USA; Department of Biology, Brigham Young University, Provo, UT 84602, USA; Department of Biology, Brigham Young University, Provo, UT 84602, USA

**Keywords:** floral traits, QTL study, Gilia, Polemoniaceae

## Abstract

Speciation is a complex process typically accompanied by significant genetic and morphological differences between sister populations. In plants, divergent floral morphologies and pollinator differences can result in reproductive isolation between populations. Here, we explore floral trait differences between two recently diverged species, *Gilia yorkii* and *G. capitata*. The distributions of floral traits in parental, F1, and F2 populations are compared, and groups of correlated traits are identified. We describe the genetic architecture of floral traits through a quantitative trait locus analysis using an F2 population of 187 individuals. While all identified quantitative trait locus were of moderate (10–25%) effect, interestingly, most quantitative trait locus intervals were non-overlapping, suggesting that, in general, traits do not share a common genetic basis. Our results provide a framework for future identification of genes involved in the evolution of floral morphology.

## Introduction

The color and dimensions of floral organs vary naturally within plant populations, facilitating adaptive change to different selective pressures (Sapir *et al.* 2021). Divergence in floral traits (e.g. color and size of petals, anther and style lengths, and throat length) makes important contributions to reproductive isolation and speciation (Rieseberg *et al.* 2006). Divergent floral morphologies can arise from pollinator-driven selection (Brothers *et al.* 2013; Wessinger *et al.* 2014; Campitelli *et al.* 2018; Kostyun *et al.* 2019; Chen *et al.* 2020), breeding system incompatibilities (Eckert *et al.* 1996; Goodwillie *et al.* 2006; Mertens *et al.* 2018; Kostyun *et al.* 2019; Roux and Pannell 2019), and random genetic drift (Tremblay and Ackerman 2001; Yoshida *et al.* 2008; Roux and Pannell 2019). Despite the central role floral morphology plays in plant evolution and taxonomy, surprisingly little is known about the genetic mechanisms that underlie the morphological evolution of flowers.

In order to understand the genetic architecture of divergent floral morphologies, quantitative trait locus (QTL) analysis of floral traits can be applied to hybrid populations. This approach uses recombination events resulting from a cross of parents that differ in heritable traits of interest to correlate phenotypes with genotypes and determine the number and effect size of loci regulating those traits (Miles and Wayne 2008). High levels of genetic and morphological variation are ideal for QTL mapping and can be obtained by crossing highly divergent parental lines. Within plants, wide crosses between morphologically distinct species often produce hybrids, such as in grasses (Freeling 2001), *Mimulus* (Bradshaw *et al.* 1995), *Aquilegia* (Hodges *et al.* 2002), and *Epidendrum* (Pinheiro *et al.* 2010). A common negative trade-off to these wide crosses is reduced fertility of the hybrid, compromising the ability to create mapping populations. However, when fertile hybrid progeny can be produced, fine-mapping of QTLs has provided valuable insights into the genes and polymorphisms that underlie morphological evolution (Ballerini *et al.* 2020; Liang *et al.* 2023).

In this study, we report a fertile inter-specific cross within the *Gilia* genus that facilitates the mapping of divergent floral morphologies. The leafy-stemmed gilias (*Gilia* section *Gilia*, Polemoniaceae) comprise 11 species found in North and South America. They are annual plants with small white or purple flowers and a raceme or panicle inflorescence (Grant 1966; Porter 2012). Hybridization between (and within) leafy-stemmed gilia species was explored by Verne Grant in the 1950s (Grant 1949), revealing weak to strong barriers to reproduction existing between species and, in some cases, between populations of the same species (Grant 1966). Subsequent to the initial biosystematic work of Grant, a new leafy-stemmed gilia species, *G. yorkii*, was discovered in 1998 (Shevock and Day 1998). A molecular phylogeny showed that *G. yorkii* is closely related to *G. capitata* (Johnson and Porter 2017). Previously, we showed that certain accessions of *G. capitata* produce fertile hybrids when crossed to *G. yorkii* (Jarvis *et al.* 2022), opening the door for an inter-species QTL analysis of floral traits. *G. yorkii* and *G. capitata* differ in numerous floral traits including flower color (Jarvis *et al.* 2022), size, stamen exsertion, and pedicel length. The inter-fertile parents differ in mating systems as well—*G. capitata* is self-incompatible, whereas *G. yorkii* is self-compatible. Both species are diploid annuals with simple growth requirements, making them convenient for genetic analysis. Because of their ease of cultivation, crossing compatibility, and divergent morphology, *G. yorkii* and *G. capitata* represent an ideal system to probe genetic causes of inter-species variation in floral traits.

Here we report floral morphology QTLs that distinguish *G. yorkii* and *G. capitata* by creating an inter-specific F2 mapping population. We find 20 QTLs linked to 17 different traits, almost all of which are non-overlapping. Considering that we also find strong morphological correlations between groups of traits, this suggests that these correlations between traits are either due to linkage disequilibrium between QTLs, or dependent on numerous shared small-effect QTLs that are difficult to detect. This study adds to the growing body of literature documenting QTLs for floral trait differences between species and provides a framework for future identification of the underlying genes.

## Materials and methods

With the exception of plants grown for initial measurements of floral traits in the parent lines (see below), all plant materials, mapping populations, DNA isolation, sequencing, genome assembly, annotation, genotyping-by-sequencing, and genetic map construction were all performed as outlined in Jarvis *et al.* (2022).

### Plant growth conditions

*G. yorkii* and *G. capitata* plants were grown indoors in a growth room prior to growing the mapping population to get initial trait measurements. These plants were grown in Sungro soilless potting mix supplemented with 18 g/l osmocote in 6-in. pots under 16-h days using fluorescent lights, with a constant temperature of 20∘C.

### Floral traits

**Fig. 1.**
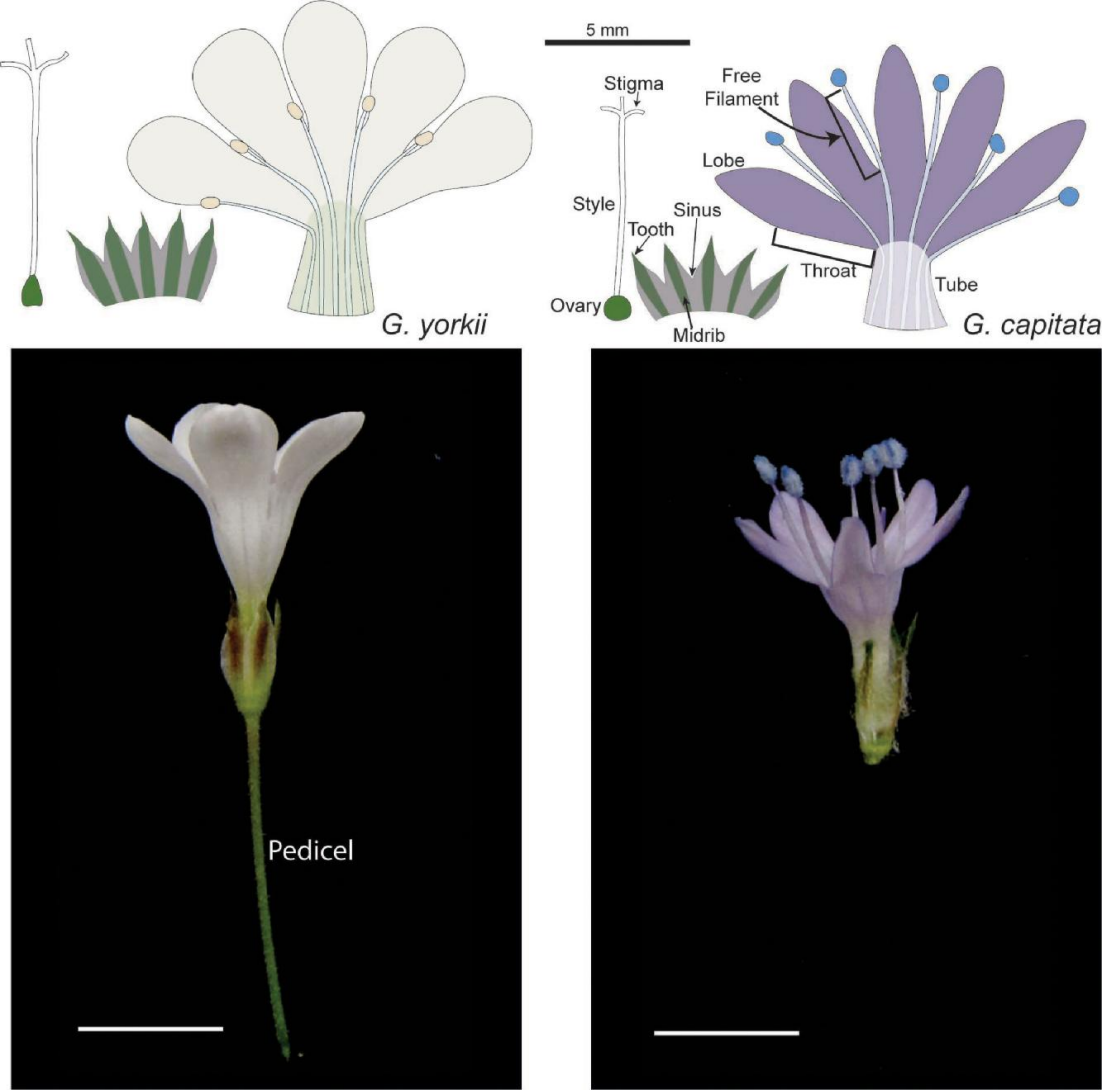
Typical flowers of *G. yorkii* (left) and *G. capitata* (right). Floral organs corresponding to traits included in the QTL analysis are labeled.

Floral traits were measured digitally from images of dissected, fresh flowers. For the first growth room measurements, 30 flowers were sampled from 5–6 individuals of each species. For the greenhouse, 1–2 flowers were sampled from each individual in the F2 population, and 1–3 flowers were sampled from each individual in the parent and F1 populations. Flowers were cut horizontally at the base to separate the calyx, corolla, and ovary from the pedicel. The calyx was slit from a sinus to the base. The corolla was slit from the base of the corolla tube to the sinus of petal lobes, taking care to be on one side of the free filament of the corresponding stamen. Corolla and calyx were laid on a glass microscope slide coated with double-sided tape. Images were taken with a Leica s8apo dissecting scope. All images were then processed using the Leica Application Suite X (LASX) software using the measuring tool, and all measurements were recorded in an Excel spreadsheet. Measured floral traits were divided into four categories: corolla, reproductive, sepal, and other traits. A full list of all traits is found in Table 1. A diagram including the major floral parts is found in Fig. 1.

**Table 1.** Mean trait values and standard errors for *G. yorkii*, *G. capitata*, F1, and F2 populations, *T*-test *P*-values between parent values, broad-sense heritability ($H^{2}$), and Shapiro–Wilks normality test of the distribution (^**^0.01 significance level; ^***^0.001; ^****^0.0001; n.s. means the distribution is not significantly different from normal).

Trait	*G. yorkii*	*G. capitata*	F1	F2	*P*-value	Heritability	Normality
Petal length	9.28±1.16	9.45±0.42	9.23±0.50	8.51±1.29	0.731	0.85	**
Petal lobe length	6.12±0.86	6.01±0.19	5.85±0.34	5.47±1.03	0.751	0.89	**
Petal lobe width	2.38±0.27	1.89±0.24	1.99±0.13	1.87±0.37	0.002	0.87	n.s.
Petal tube length	3.11±0.50	3.30±0.37	3.37±0.30	2.94±0.43	0.409	0.52	n.s.
Petal tube width	2.53±0.21	3.32±0.44	2.89±0.26	2.87±0.37	0.000	0.53	n.s.
Throat length	2.11±0.34	2.19±0.18	2.12±0.16	1.90±0.50	0.558	0.89	**
Filament length	5.70±0.90	8.20±0.74	6.96±0.52	5.68±1.41	0.000	0.87	***
Free filament length	0.52±0.15	2.83±0.36	1.55±0.26	1.21±0.98	0.000	0.93	****
Anther length	0.78±0.09	0.84±0.06	0.83±0.08	0.78±0.13	0.205	0.55	**
Anther width	0.61±0.08	0.77±0.08	0.68±0.08	0.60±0.16	0.001	0.79	n.s.
Style length	6.34±0.60	6.71±0.75	6.37±0.39	6.02±1.17	0.309	0.89	n.s.
Stigma length	1.08±0.12	0.61±0.05	0.85±0.15	0.86±0.20	0.000	0.42	***
Ovary shape	0.78±0.06	0.92±0.09	0.77±0.06	0.79±0.08	0.002	0.30	n.s.
Sepal length	3.58±0.49	2.99±0.46	3.68±0.37	3.36±0.49	0.028	0.44	n.s.
Sepal sinus length	1.75±0.32	1.64±0.21	1.87±0.22	1.79±0.32	0.463	0.53	n.s.
Sepal tooth length	1.76±0.29	1.34±0.32	1.79±0.17	1.53±0.30	0.013	0.68	n.s.
Sepal midrib width	0.42±0.05	0.40±0.08	0.41±0.05	0.40±0.06	0.512	0.30	n.s.
Pedicel length	16.60±11.89	0.75±0.36	1.41±0.41	2.96±3.46	0.041	0.99	****
Internode length	17.00±8.00	0.33±0.21	1.09±0.56	2.66±3.47	0.010	0.97	****
Days to flower	66.67±9.77	56.65±6.98	62.81±7.58	54.55±8.84	0.017	0.26	****
Vegetative rosette diameter	15.69±3.70	16.25±2.29	21.58±3.64	22.33±4.02	0.678	0.18	n.s.

Traits are ordered by group (corolla, reproductive, calyx, and other traits) for ease of reference. All measurements are in millimeters (mm).

Data for the Procrustes analysis of floral shape were collected in ImageJ by placing landmark points at the corners of the opened floral tube, the sinus of each petal lobe, two points at the widest part of each petal lobe, and one point at the tip of the petal lobe.

### Statistical analysis

Mean values, standard error, and Student’s *t*-test between parent values were all calculated in R using base functions. Broad-sense heritability ($H^{2}$), or the proportion of variance not due to environmental factors alone, was calculated manually in Excel using the formula


(1) $$H^{2}=\frac{V_{F2}-V_{F1}}{V_{F2}},$$

where $V_{F1}$ and $V_{F2}$ are the variances of the F1 and F2 populations, respectively.

Trait histograms were generated in R using the “ggplot2” package. Correlations between traits were calculated using Pearson’s correlations in R, and the correlation plot was generated using the “corrplot” package. Procrustes analysis and subsequent principal components were generated using the “procGPA” function from the “shapes” package. QTL data were loaded and analyzed in the “rqtl” package (Broman *et al.* 2003; Broman and Sen 2009). Single-QTL mapping was performed with the “scanone” function using the extended Haley–Knott regression mapping function. Composite interval mapping (CIM) was performed using the “cim” function, also using the extended Haley–Knott regression mapping function. A total of 10% logarithm of the odds (LOD) significance levels for mapping results were determined by a permutation test of 40,000 permutations per trait for single-QTL mapping, and 20,000 permutations per trait for CIM. QTL intervals were declared from the CIM mapping results using the “lodint” function using a LOD drop of 1.5 and the position of the qtl index corresponding to the marker with the highest LOD in intervals that extended above the significance threshold. Percent variance explained (PVE) was calculated using the equation below:


(2) $$\mathrm{PVE}=1-10^{-(2/n)*\mathrm{LOD}},$$

where *n* is the number of individuals (n≤187) and LOD is the highest LOD score for each trait across all markers.

## Results

### Floral morphology differences between *G. capitata* and *G. yorkii*

The overall floral morphology of *G. capitata* and *G. yorkii* is similar and shared with diverse *Gilia* species and many related Polemonicaceae (Fig. 1). The sepals are united at the base with a free toothed apex and a distinct green (occasionally infused with purple) band running along the midrib flanked by a light-green to hyaline margin. Like the sepals, petals of both species are also fused basally, and this fused region can be divided into a narrow basal tube which transitions into a flaring throat. Distal to the throat, five unfused lobes are symmetrically arranged. Five stamens alternate with the petals, and the stamen filaments are adnate with the basal petal along both the tube and throat, and then extend slightly on a free filament between petal lobes. The pistil is composed of a distinct round to oval-shaped ovary at the base and an elongated style which branches apically into three distinct stigmas.

Within this shared floral groundplan, multiple differences across *G. capitata* and *G. yorkii* are apparent. *G. capitata* flowers are notably smaller with exserted stamens, narrower petal lobes, and a hardly perceptible pedicel compared to the larger *G. yorkii* flowers with inserted stamens, wide petal lobes, and a long pedicel. To identify consistent morphological differences between the parent species, an initial study of floral traits was conducted on plants grown in a growth room, with trait means described in Supplementary Table S1. Significant differences were found for all sepal traits except for sepal tooth length, with *G. yorkii* being the larger species. Similarly, in the petal whorl *G. yorkii* had significantly larger petal length, petal lobe length, tube length, throat length, and petal lobe width. Petal tube width was also significantly different, but in this case *G. capitata* was larger. In the stamen whorl, filament, free filament length, and anther width were all significantly larger in *G. capitata*. In the pistil whorl, the ratio of ovary width to length, a measure of ovary shape, was significantly larger in *G. capitata*. While there was no significant difference in style length, stigma length was significantly larger in *G. yorkii*. With the exceptions of stigma length, ovary shape, sepal length, sepal midrib width, and vegetative rosette diameter, most traits had relatively high broad-sense heritabilities ($H^{2}>0.5$), showing that most of our measured traits are good candidates for QTL analysis.

For most traits measured, the variance was larger in *G. capitata* than in *G. yorkii*. While *G. yorkii* is self-compatible and a highly inbred line was used for all measurements, the self-incompatible *G. capitata* was sib-crossed for five generations, and thus is significantly less inbred than *G. yorkii*. It is likely that some of the additional variance in the *G. capitata* floral traits are a result of residual segregating genetic variation.

### Distribution of floral traits in an F2 mapping population

Floral traits were measured on *G. yorkii* and *G. capitata* in two similar environments: an indoor growth room and a greenhouse. Unexpectedly, several petal length measurements that were significantly different in the growth room environment were no longer significant in the greenhouse environment, which may suggest a G×E interaction for petal traits. This may also be due to the reduced number of individuals measured per parent population in the greenhouse as compared to the growth room study, translating to reduced statistical power in the greenhouse populations. Beyond this, most of the remaining traits had similar significance levels across the two environments, exhibiting stable differences between *G. yorkii* and *G. capitata*.

Normality of the floral traits in the F2 population was measured using the Shapiro–Wilk test. Of the 17 floral traits and 4 additional traits, 11 show normality at the α=0.01 level, and are indicated by n.s. in Table 1. For the other 10 traits, upon visual inspection, it was determined that free filament length, internode length, and pedicel length have the most severely skewed distributions (Supplementary Fig. S1), and a log-transformed phenotype of these traits was included in the QTL analysis in addition to the original phenotype (Goh and Yap 2009). Of these skewed distributions, free filament length was the only trait where *G. capitata* had a higher mean value than *G. yorkii*.

Broad-sense heritability of the floral traits ranges between 0.30 and 0.99. While many of the floral traits have high heritability values ($H^{2}>0.6$), some traits have lower heritabilities. In particular, petal tube length, petal tube width, anther length, stigma length, sepal sinus length, and sepal midrib width have heritabilities ranging from 0.30 to 0.55 (Table 1). It is not immediately apparent why these traits, which are spread across the floral whorls, have low heritabilities compared to the other traits, although it does limit their potential as traits to follow up on in further genetic analyses. Two other traits, days to flower and vegetative rosette diameter, show extremely low heritability values of 0.26 and 0.18, respectively. These traits are expected to be highly sensitive to environmental conditions, and this is confirmed by the observation that the F1 and F2 populations have similar variances for these traits.

### Trait correlations

**Fig. 2.**
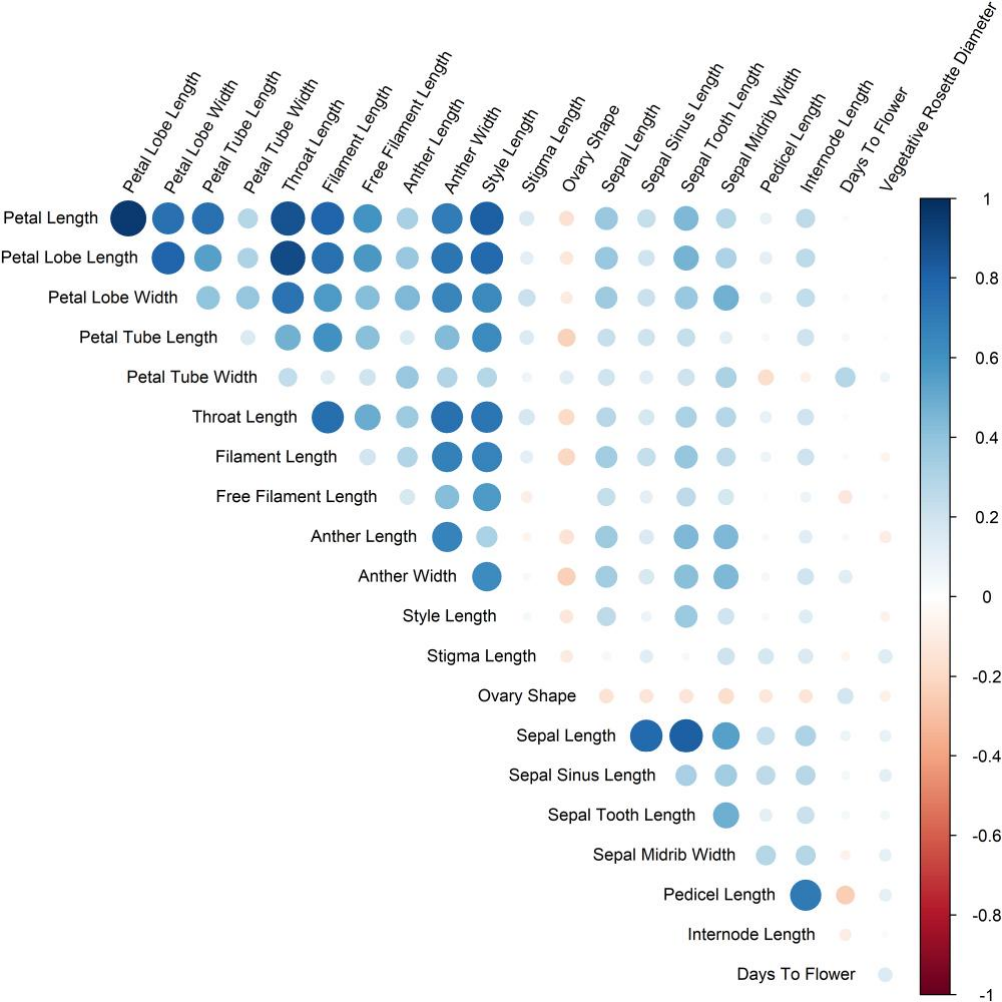
Correlations between floral traits. Color represents the direction of correlation, whereas intensity and circle size represent the degree of correlation.

To better understand how individual floral traits are connected, we calculated Spearman’s correlations and identified groups of correlated traits for all floral traits. Figure 2 shows correlations between all floral traits. The largest group of correlated traits consisted of petal traits (petal length, lobe length, and lobe width), anther width, and style length, all of which showed strong positive correlations with each other. Unexpectedly, anther length did not strongly correlate with traits in this group besides anther width. In addition, petal tube length, petal tube width, and free filament length correlated with one or more, but not all, traits in this group. Calyx measurements comprised a second distinct group of correlated traits. Sepal length showed strong correlations with all other calyx traits, while correlations between sepal midrib width, sepal tooth length, and sepal sinus length by themselves were low to moderate. Internode length and pedicel length, both in florescence architecture traits, were strongly correlated with each other. Vegetative rosette diameter and days to flower did not correlate significantly with any floral morphological traits (Fig. 2).

### Morphometric analysis

**Fig. 3.**
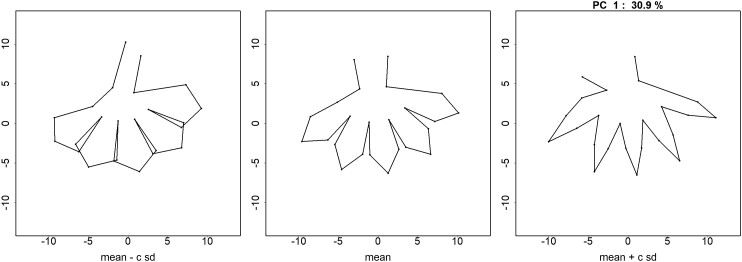
Principal component 1 from PCA analysis of Procrustes-adjusted landmark data. The left and right boxes represent one standard deviation below and above the mean of PC1, and capture the major differences between *G. yorkii* and *G. capitata* flowers. PC1 explains 30.9% of the phenotypic variation in the combined parent populations.

To investigate whether changes in overall floral morphology are significantly different between the parent populations, we collected landmark data points from whole flowers for a Procrustes analysis, which uses a principal component analysis (PCA) to reduce the dimensionality of the data. The first principal component (Fig. 3) accounts for 30.9% of the variance present in the combined parent population. Visually, PC1 captures much of the variation we observed previously between *G. yorkii* and *G. capitata* flowers, where *G. yorkii* has wider petal lobes and a longer floral tube as compared to *G. capitata*, and it effectively separates *G. yorkii* and *G. capitata* individuals. When F2 landmark data are transformed into this principal component background, they generate intermediate values (Supplementary Fig. S2). QTL mapping of the F2 PC1 values resulted in no significant QTLs, suggesting that overall floral shape is dependent on many small-effect QTLs, rather than major effect QTLs.

### QTL analysis

**Fig. 4.**
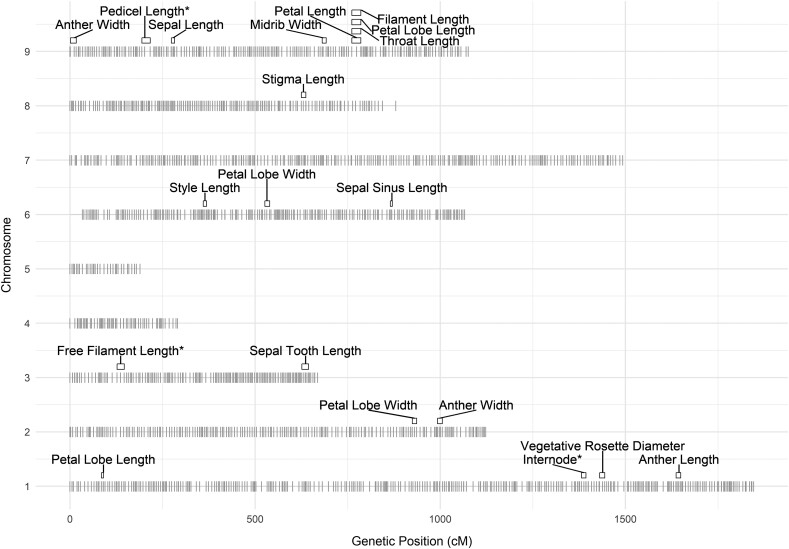
QTL intervals for all significant traits. Marker distribution is shown in gray along each chromosome. Rectangles represent 1.5 LOD drop intervals with right and left endpoints shown. Trait names are shown above their respective QTL(s). Three trait QTLs denoted with an asterisk (free filament length, pedicel length, and internode length) were considered for log transformation of the raw phenotype data. For pedicel length, only the log-transformed data produced a significant QTL. For free filament length and internode length, only the raw phenotype data produced significant QTLs.

Using our high-density genetic map of 5,335 markers, we conducted single-QTL mapping and CIM for all 21 traits. Single-QTL mapping resulted in extremely large confidence intervals (Supplementary Figs. S3–S8), whereas CIM resulted in clearly defined and separate confidence intervals for many traits. A total of 20 significant QTL were identified across all floral and other traits from the CIM results. QTLs were found on six out of the nine chromosomes present in *Gilia* (Fig. 4 and Table 2). Fourteen traits had one significant QTL each and three traits (petal lobe length, petal lobe width, and anther width) mapped to two QTLs each.

**Table 2.** List of traits with significant QTL.

Trait	Chr	Left	Pos (cM)	Right	Peak LOD	PVE
Petal length	9	761.4	774.5	785.6	7.2	16.3
Petal lobe length	1	84.8	90.3	94.9	6.2	14.1
Petal lobe length	9	761.4	774.5	785.6	7.0	15.8
Petal lobe width	2	924.8	930.9	936.3	7.0	15.8
Petal lobe width	6	526.4	531.9	539.7	6.2	14.2
Throat length	9	761.4	774.5	785.6	6.3	14.3
Filament length	9	761.4	774.5	785.6	7.6	17.1
Free filament length	3	127.2	136.7	146.5	10.1	22.0
Anther length	1	1,637.2	1,645.0	1,650.0	7.4	16.6
Anther width	2	992.9	997.8	1,006.0	5.8	13.4
Anther width	9	1.1	11.7	17.2	7.4	16.6
Style length	6	360.0	364.7	369.0	6.1	13.9
Stigma length	7	625.0	630.5	637.4	6.2	14.1
Sepal length	9	273.8	278.4	282.7	6.7	15.3
Sepal sinus length	6	866.7	869.4	877.2	7.6	17.0
Sepal tooth length	3	626.3	636.1	644.5	5.7	13.1
Sepal midrib width	9	681.5	683.9	691.9	9.9	21.5
Internode length	1	1,381.4	1,388.3	1,393.8	10.3	22.4
Pedicel length	9	194.3	202.1	216.6	9.9	21.6
Vegetative rosette diameter	1	1,430.5	1,438.8	1,444.8	7.7	17.3

Intervals were calculated using a 1.5 LOD drop. Left and right endpoints, position of the highest-correlated marker, and PVE are shown. Traits are ordered by group (corolla, reproductive, calyx, and other traits) for ease of reference.

Notably, eight of the 20 QTLs localize to chromosome 9, including several highly correlated corolla length traits (petal length, petal lobe length, throat length, and filament length), as well as anther width, pedicel length, sepal length, and sepal midrib width (Fig. 4 and Table 2). Although the single-QTL mapping results were nearly entirely overlapping, the CIM results resolved nearly all traits into separate QTLs, showing low genetic overlap between traits overall.

QTLs were found on chromosomes 1, 2, 3, 4, 6, 8, and 9, but were absent on chromosomes 4, 5, and 7. For chromosome 7, it appears that no floral trait QTLs are present on this chromosome. For chromosomes 4 and 5, the selected markers do not span the entire length of the chromosome due to excessive segregation distortion of markers mapping to these chromosomes. It is possible that there are additional QTLs in the regions not covered by our marker set, which would be undetectable by our analysis.

All identified QTLs had effects ranging from 10% to 25% of variance (PVE) explained in the F2 population. The four traits with the highest PVE values are internode length (22.4%), free filament length (22.0%), pedicel length (21.6%), and sepal midrib width (21.5%) (Table 2). Internode length, free filament length, and pedicel length also showed the highest heritability within the F2 population, with $H^{2}$ values of 0.97, 0.93, and 0.99, respectively. Sepal midrib width, on the other hand, had low heritability in the F2 with an $H^{2}$ value of 0.30. This indicates that, although heritability is low, most of the genetic variance available for sepal midrib width is captured by a single QTL.

## Discussion

In this study, we have described floral trait averages, correlations, and QTLs from an F2 population derived from a cross between *G. yorkii* and *G. capitata*. These two species are divergent for floral color and inflorescence architecture, which have been described in a previous publication (Jarvis *et al.* 2022). Here, we explore the genetic structure of quantitative floral traits that distinguish *G. yorkii* and *G. capitata*. We find that nearly all trait QTLs occupy unique positions across chromosomes. The only exception to this is the colocalization of petal length, petal lobe length, throat length, and filament length on chromosome 9. This case is unsurprising, considering these correlated traits likely reflect a common developmental origin. The separation of trait QTLs throughout the genome is in contrast to other QTL studies in plant species, where some colocalization of QTLs is observed, due to either multiple linked loci or pleiotropic loci (Bouck *et al.* 2007; Wessinger *et al.* 2014; Zhu *et al.* 2014; Kostyun *et al.* 2019). Our findings suggest that most floral trait differences between *G. yorkii* and *G. capitata* are controlled by unique genetic loci, and are thus likely to be regulated by distinct genetic mechanisms. Thus, divergent floral morphologies between the two species are likely to have been under less genetic constraints with regard to individual trait changes.

Although most QTLs occupy distinct chromosomal regions, strong morphological correlations appear to be present within *Gilia* flowers. We found two clear groups of strongly correlated traits within the F2 population: one consisting of corolla traits and style length and the other consisting of sepal traits. Interestingly, some traits have a clear lack of correlation with any other traits. Anther length, despite having a strong correlation with anther width, shows only weak correlations with other corolla traits. This contrasts with reported studies in *Mimulus* (Fenster and Ritland 1994; Fishman *et al.* 2002; Hall *et al.* 2006; Fishman *et al.* 2015) that have higher correlations of anther length with other corolla traits, suggesting a weaker connection of anther length and corolla traits within *Gilia* species. Vegetative rosette diameter shows strikingly low correlations with all other traits, showing that floral trait correlations are not biased by the plant’s overall size. Stigma length was previously shown to be connected to calyx pubescence, calyx lobe reflexion, and capsule dehiscence in intraspecies crosses between *G. capitata* subspecies (Grant 1950). Our results agree with that study, in that stigma length has a weak association with petal lobe width. In summary, most of the corolla and sepal traits correlate strongly within, but not across, their respective floral whorls. Some traits, like stigma length, do not seem to be connected directly to any other traits, even with physically adjacent traits.

While many traits showed high broad-sense heritability between the parent and F1 generations (Table 1), the variance explained by the discovered QTLs (Table 2) is significantly lower. For example, free filament length shows a heritability of 0.93, while the single QTL discovered for this trait explains 22.0% of the variance within the F2 population. Part of this discrepancy may be due to physical chromosomal regions not included in the genetic map, especially on chromosomes 4 and 5. Any causative QTLs located within these regions are not detectable by our QTL analysis. Another possibility is that some of the trait variation consists of small-effect loci that do not pass the significance threshold. Other traits have significantly more agreement between their heritability and percent of phenotypic variance explained in the F2 population. For example, vegetative rosette diameter has a heritability of 0.18 and its single QTL explains 17.3% of the variation, indicating that the genetic variance is nearly fully explained by a single QTL for this trait. Similarly, sepal midrib width has a heritability of 0.30 and its QTL explains 21.5% of the variation. Overall, most of the traits examined appear to have genetic variation beyond that explained by the discovered QTLs, whereas two traits, vegetative rosette diameter and stigma length, appear to have a majority of the phenotypic variance explained by a single QTL.

The adaptive purpose, if indeed one exists, for the morphological differences of *G. yorkii* and *G. capitata* flowers is still unclear. Considering the importance of the size and shape of floral organs for successful pollination, it seems likely that there may be pollinator differences between these species. Verne Grant documented the potential pollinators of *G. capitata* and some of its subspecies (Grant V and Grant KA 1965), which attract a wide range of insect visitors, including various bees, beeflies, beetles, and butterflies (Grant V and Grant KA 1965). Since the discovery of *G. yorkii*, its pollinators have not yet been reported. Comparing *G. yorkii* to other species within Polemoniaceae that share a similar floral shape and inflorescence structure, such as *G. achilleifolia *ssp.*multicaulis*, *G. angelensis*, and *G. tricolor*, it is likely that pollinator classes for *G. yorkii* would be similar to *G. capitata* (i.e. bees, beeflies, beetles, and butterflies), but may be smaller in size overall (Grant V and Grant KA 1965). The apparent difference in breeding systems may also help explain some of the trait variation. The reduction in stamen exsertion and lack of pigmentation in *G. yorkii* could be consistent with some level of self-pollination. However, self-incompatibility is not fixed across populations of *G. capitata* (Grant 1950), and we have identified some self-compatible populations that can self-fertilize in the greenhouse. Field observations are needed to verify these suspected pollinator attractions and any reproductive system differences.

In summary, we have presented the first study connecting the floral traits of two *Gilia* species to their genetic underpinnings. These traits are confirmed to be quantitative in nature, as evidenced by the absence of major effect QTL explaining more than 50% of phenotypic variation, and in general, trait QTLs map to separate regions across the genome. Along with this, petal and sepal traits constitute two distinct groups of correlated traits, suggesting that each group has morphological constraints despite individual traits having separate genetic bases. Future work is required to investigate candidate genes underlying the QTLs identified in this study.

## Supplementary Material

jkae106_Supplementary_Data

## Data Availability

All phenotypic and genotypic data used for the QTL analysis and morphometric analysis, as well as all data analysis scripts, are available on Github at https://github.com/detemplej/Gilia-QTL-data (DOI: 10.5281/zenodo.11506256). Original microscope images of dissected flowers are available upon request. Supplemental material available at G3 online.
