# Supplementary material for: QTL analysis of divergent floral morphology traits between Gilia yorkii and G. capitata
Source: G3 (Bethesda). 2024 May 21;14(8):jkae106. doi: 10.1093/g3journal/jkae106 (PMC11304593; doi:10.1093/g3journal/jkae106)
Supplement: jkae106_Supplementary_Data [file jkae106_supplementary_data.pdf]

| Trait | G. yorkii | G. capitata | P-value |
|---|---|---|---|
| Petal Length | 10.53 ± 0.38 | 9.61 ± 0.79 | 0.000 |
| Petal Lobe Length | 6.84 ± 0.27 | 6.44 ± 0.51 | 0.001 |
| Petal Lobe Width | 2.99 ± 0.19 | 1.80 ± 0.20 | 0.000 |
| Petal Tube Length | 3.35 ± 0.21 | 2.81 ± 0.51 | 0.000 |
| Petal Tube Width | 2.41 ± 0.23 | 3.46 ± 0.33 | 0.000 |
| Throat Length | 2.57 ± 0.18 | 2.43 ± 0.32 | 0.040 |
| Filament Length | 6.68 ± 0.21 | 7.53 ± 1.30 | 0.001 |
| Free Filament Length | 0.93 ± 0.16 | 2.44 ± 0.62 | 0.000 |
| Anther Length | 0.62 ± 0.04 | 0.64 ± 0.05 | 0.211 |
| Anther Width | 0.46 ± 0.03 | 0.56 ± 0.05 | 0.000 |
| Style Length | 7.27 ± 0.3 | 7.02 ± 0.97 | 0.185 |
| Stigma Length | 1.19 ± 0.11 | 0.63 ± 0.17 | 0.000 |
| Ovary Shape | 0.67 ± 0.05 | 0.94 ± 0.13 | 0.000 |
| Sepal Length | 2.92 ± 0.34 | 2.68 ± 0.39 | 0.014 |
| Sepal Sinus Length | 1.5 ± 0.23 | 1.23 ± 0.23 | 0.000 |
| Sepal Tooth Length | 1.34 ± 0.18 | 1.37 ± 0.21 | 0.599 |
| Sepal Midrib Width | 0.4 ± 0.04 | 0.35 ± 0.06 | 0.000 |

**Table S1** Mean trait values and standard errors for *G. yorkii* and *G. capitata* populations grown in a growth chamber, and T-test p-values between parent values. Corolla, reproductive, and calyx traits are divided within the table for ease of reference. All measurements are in millimeters (mm).

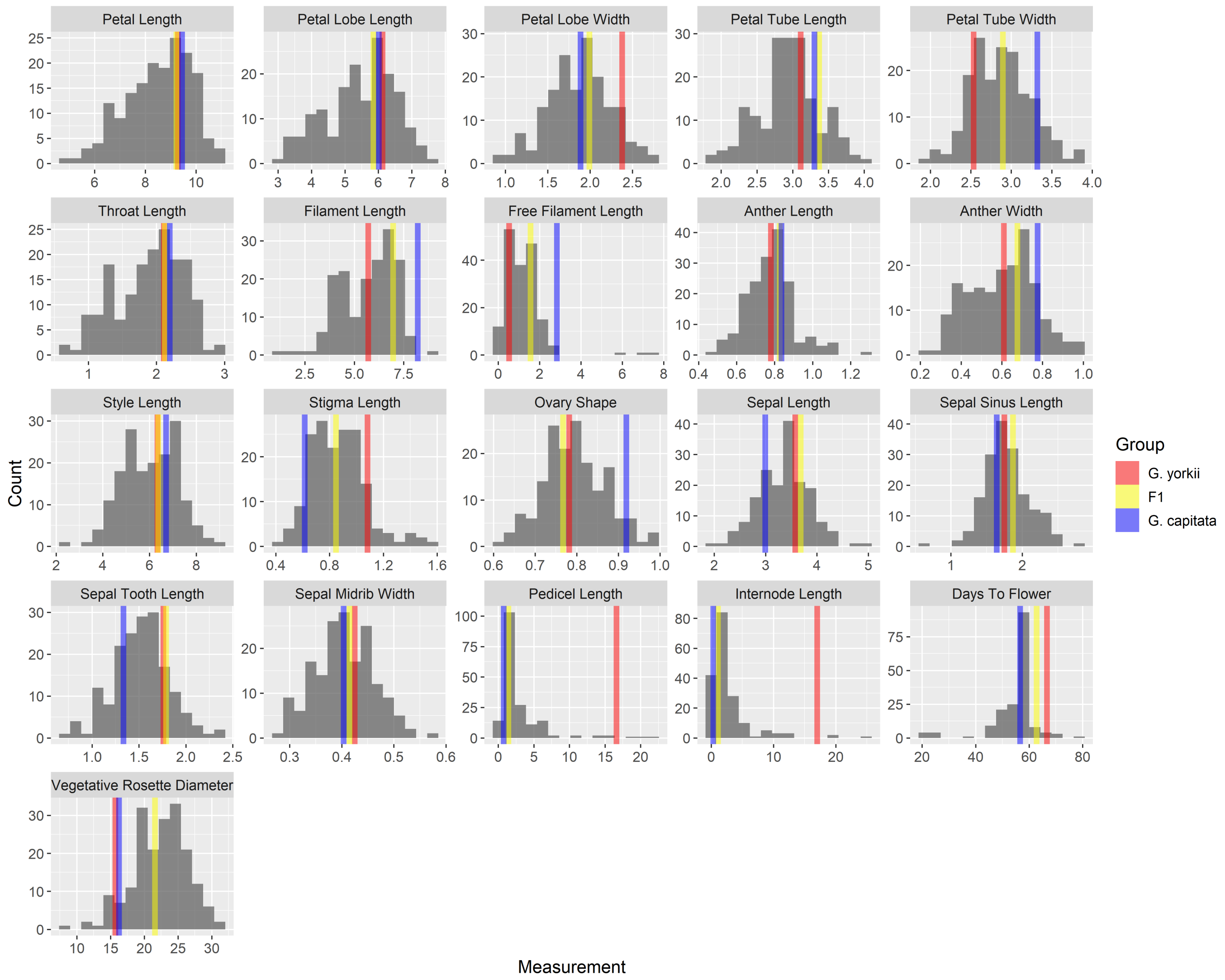


**Figure S1** Trait distributions in the F2 generation. G. yorkii, F1, and G. capitata population means are shown in red, yellow, and blue lines, respectively. Axis scales are millimeters (mm).

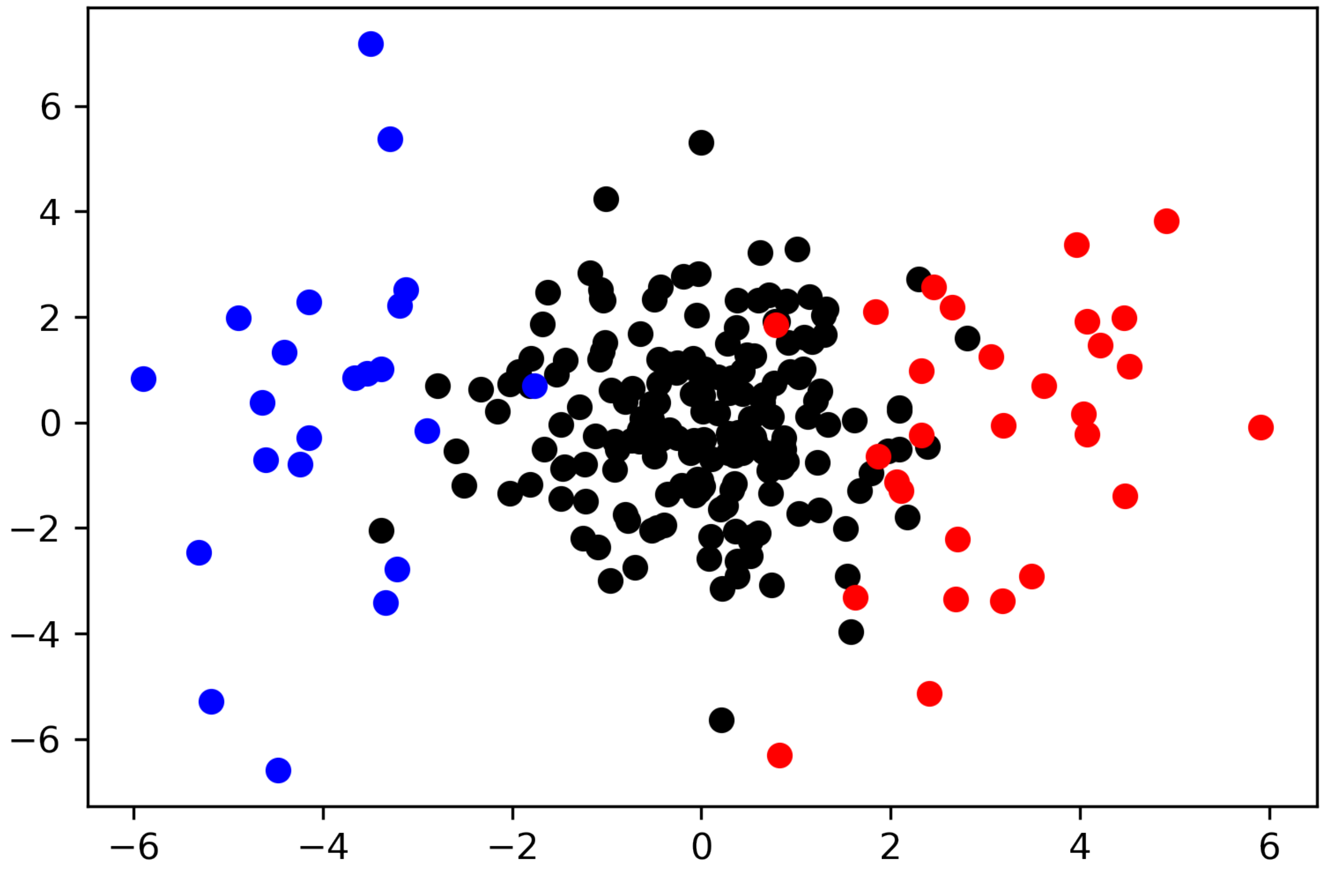


**Figure S2** F2 population projected onto PC1 (x-axis), PC2 (y-axis) space of parent populations. Each dot represents an individual plant, and the red, blue, and black colors represent *G. yorkii*, *G. capitata*, and F2 individuals respectively.

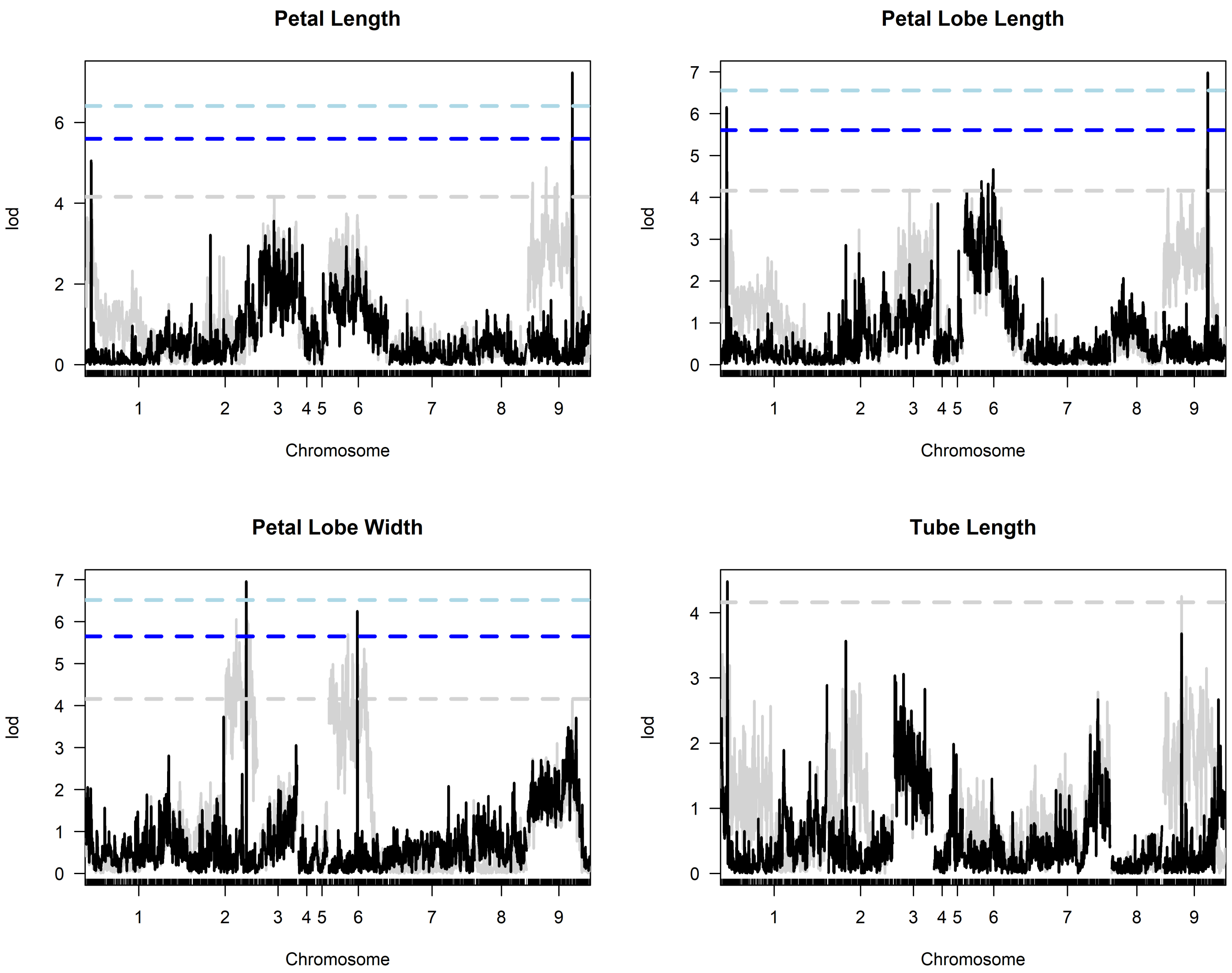


**Figure S3** QTL graphs for individual traits across all chromosomes. CIM mapping results are plotted in black, with single-QTL mapping results plotted in light gray behind. The blue and light blue dotted lines represent the 90% and 95% LOD significance thresholds, respectively, for CIM results. The light gray dotted line represents the 95% LOD threshold for single-QTL mapping results.

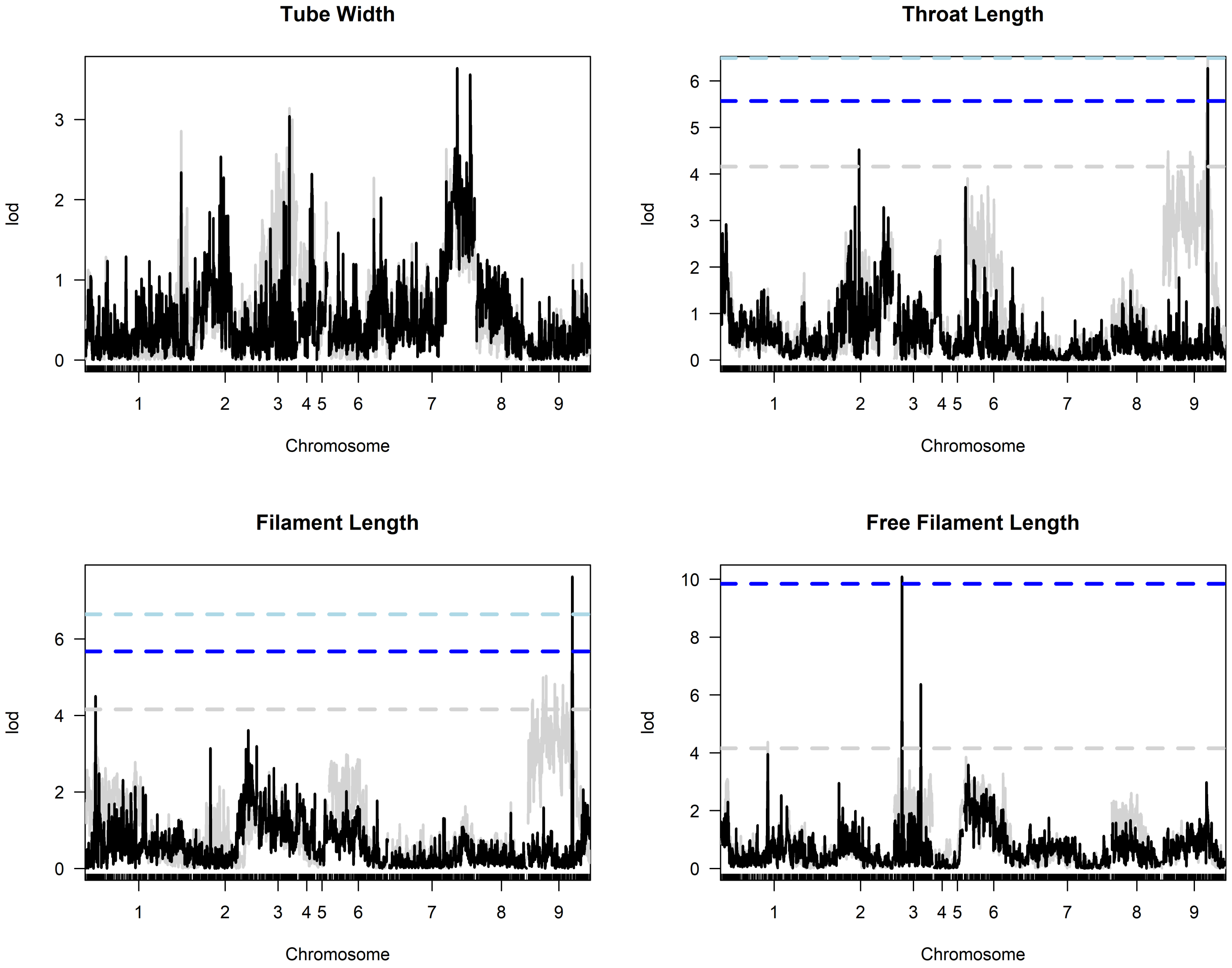


**Figure S4** QTL graphs for individual traits across all chromosomes. CIM mapping results are plotted in black, with single-QTL mapping results plotted in light gray behind. The blue and light blue dotted lines represent the 90% and 95% LOD significance thresholds, respectively, for CIM results. The light gray dotted line represents the 95% LOD threshold for single-QTL mapping results.

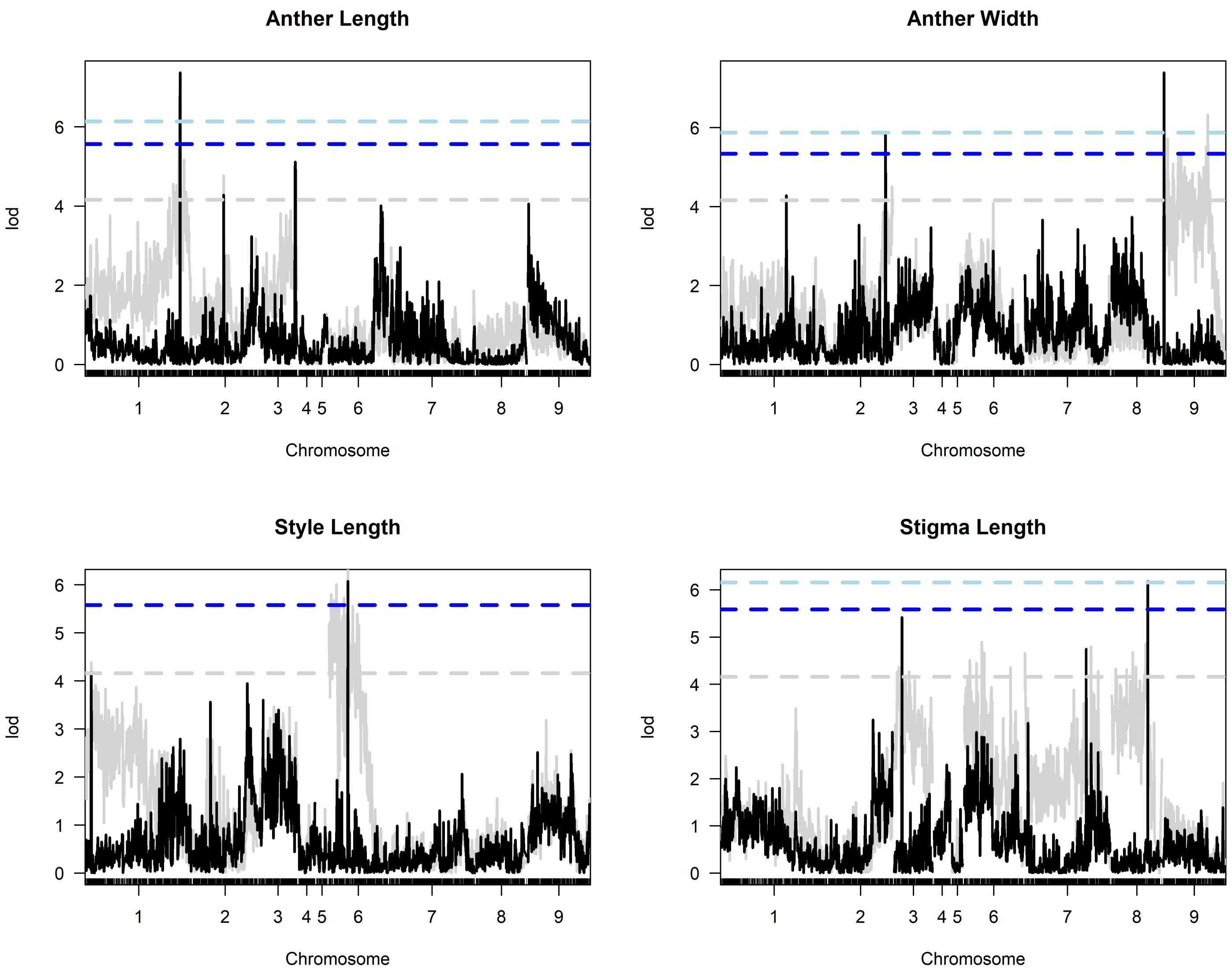


**Figure S5** QTL graphs for individual traits across all chromosomes. CIM mapping results are plotted in black, with single-QTL mapping results plotted in light gray behind. The blue and light blue dotted lines represent the 90% and 95% LOD significance thresholds, respectively, for CIM results. The light gray dotted line represents the 95% LOD threshold for single-QTL mapping results.

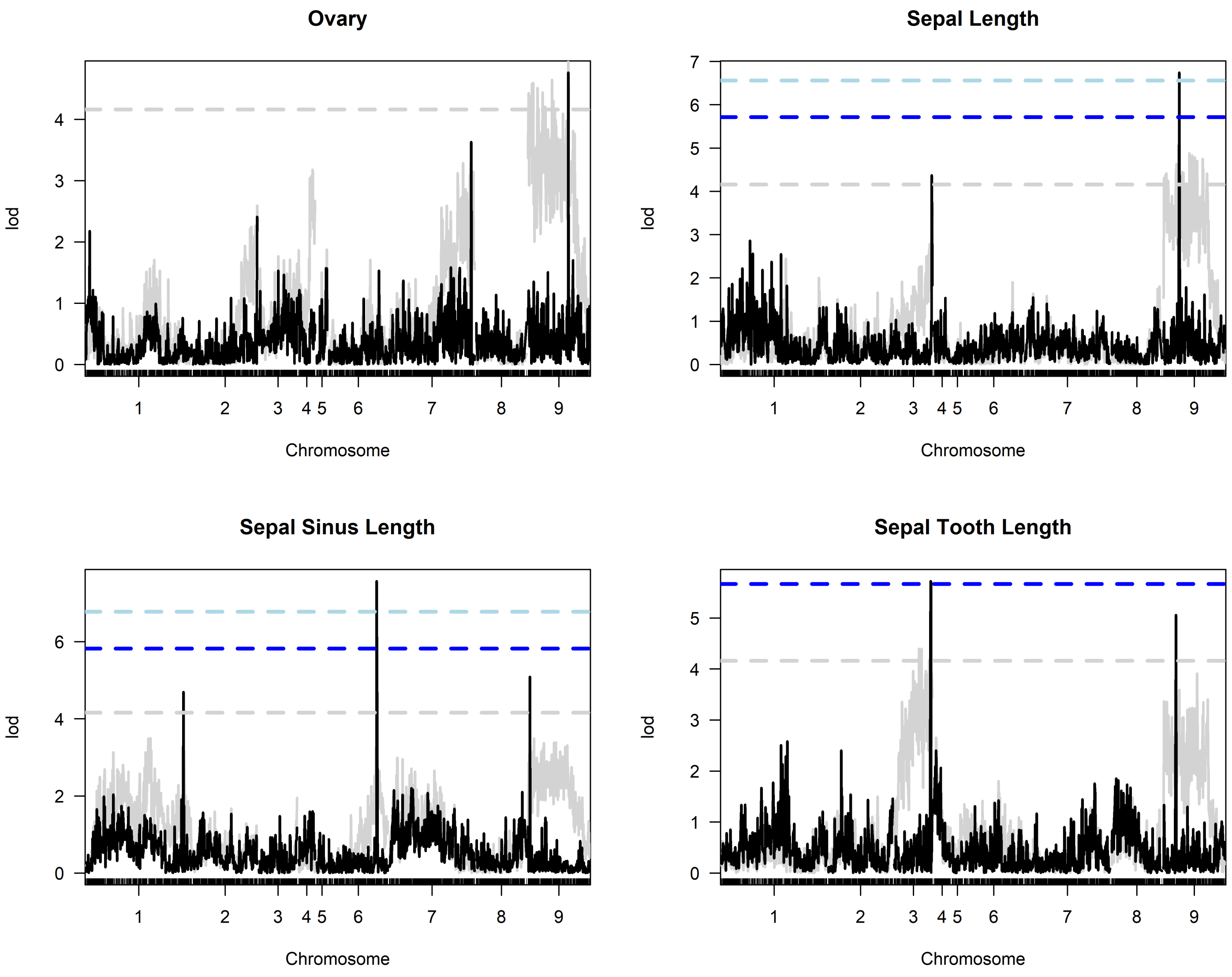


**Figure S6** QTL graphs for individual traits across all chromosomes. CIM mapping results are plotted in black, with single-QTL mapping results plotted in light gray behind. The blue and light blue dotted lines represent the 90% and 95% LOD significance thresholds, respectively, for CIM results. The light gray dotted line represents the 95% LOD threshold for single-QTL mapping results.

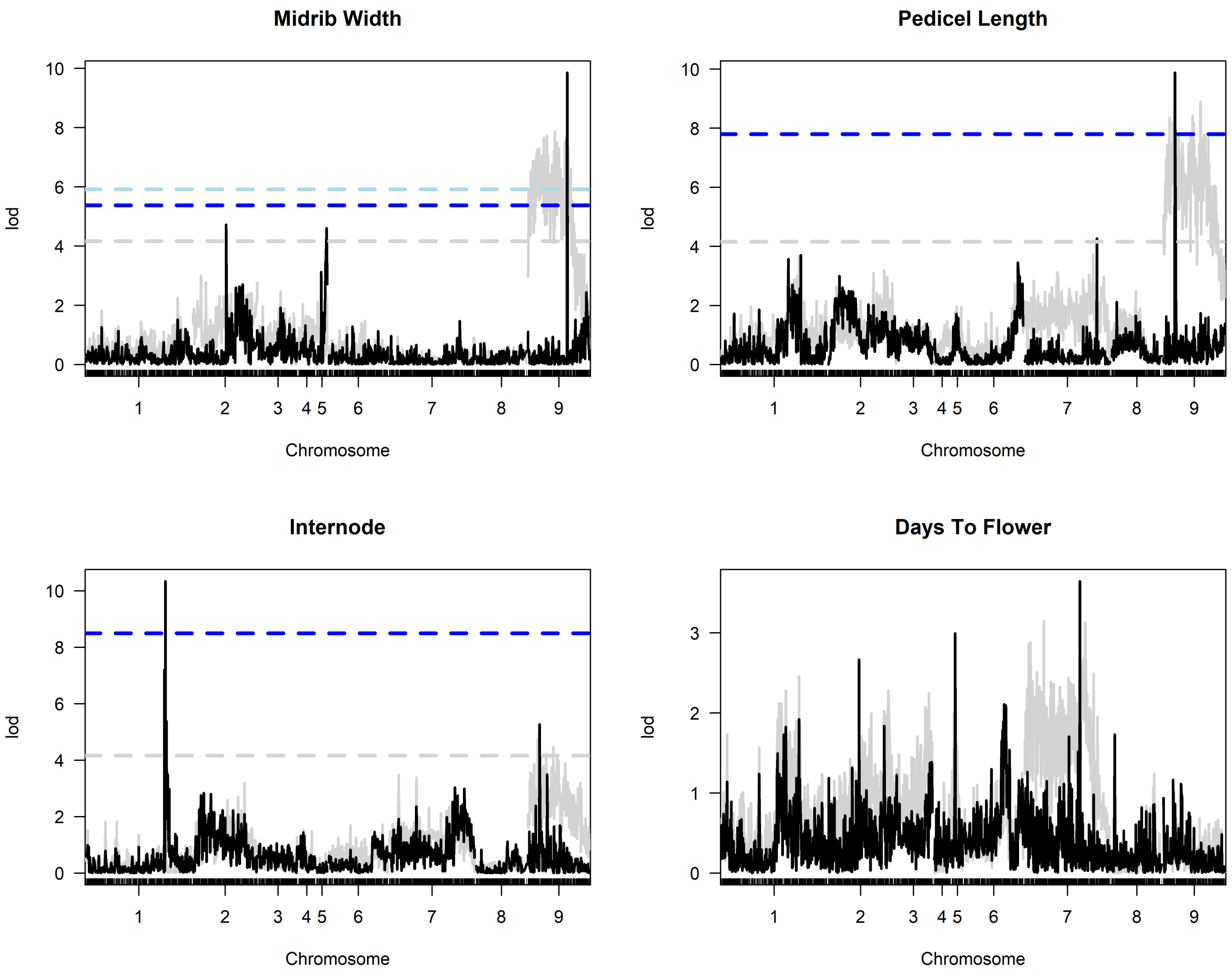


**Figure S7** QTL graphs for individual traits across all chromosomes. CIM mapping results are plotted in black, with single-QTL mapping results plotted in light gray behind. The blue and light blue dotted lines represent the 90% and 95% LOD significance thresholds, respectively, for CIM results. The light gray dotted line represents the 95% LOD threshold for single-QTL mapping results.

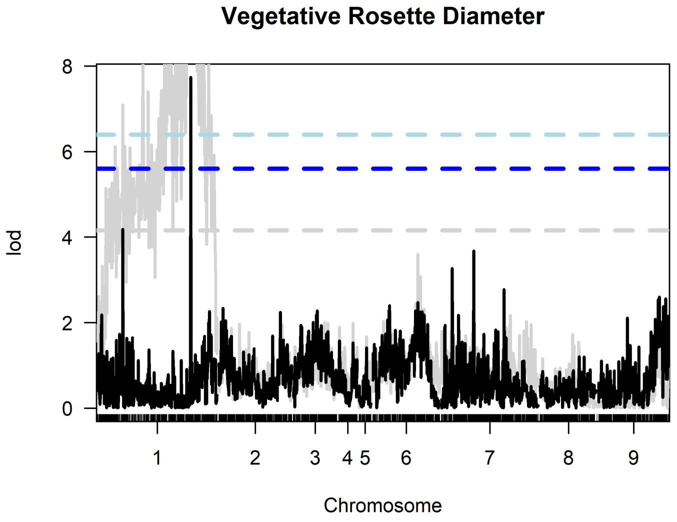


**Figure S8** QTL graphs for individual traits across all chromosomes. CIM mapping results are plotted in black, with single-QTL mapping results plotted in light gray behind. The blue and light blue dotted lines represent the 90% and 95% LOD significance thresholds, respectively, for CIM results. The light gray dotted line represents the 95% LOD threshold for single-QTL mapping results.
